# Gender-Based Differences in Psychological, Nutritional, Physical Activity, and Oral Health Factors Associated with Stress in Teachers

**DOI:** 10.3390/ijerph21040385

**Published:** 2024-03-22

**Authors:** Helia Carmen Peris-Ramos, María Carreira Míguez, Stephanie Rodriguez-Besteiro, Susana David-Fernandez, Vicente Javier Clemente-Suárez

**Affiliations:** 1 Clinical Odontology Department, Faculty of Biomedical and Health Sciences, Universidad Europea de Madrid, Tajo Street, s/n, 28670 Madrid, Spain; helia.peris@universidadeuropea.es (H.C.P.-R.); susana.david@universidadeuropea.es (S.D.-F.); 2 Faculty of Sports Sciences, Universidad Europea de Madrid, Tajo Street, s/n, 28670 Madrid, Spain; mcarreiram@hotmail.com (M.C.M.); annie310791@gmail.com (S.R.-B.); 3 Grupo de Investigación en Cultura, Educación y Sociedad, Universidad de la Costa, Barranquilla 080002, Colombia

**Keywords:** gender differences, stress, burnout, teachers, professors, psychological factors, nutrition, physical activity, oral health

## Abstract

The aim of this study was to analyze gender differences in stress-related factors among active teachers. A cross-sectional study was conducted to examine gender disparities in psychological, nutritional, physical activity, and oral health factors and how these habits correlate with stress and burnout in their work environment. The sample comprised 1037 teachers from Spain, Colombia, and Chile, consisting of 40.1% men and 59.9% women, with an average age of 41 years and teaching experience of 11.8 ± 9.2 and 12.2 ± 8.7 years, respectively. They were evaluated using a compilation of questionnaires with the objective of analyzing gender differences in habits that are associated with stress levels in teachers. The findings revealed that men had significantly higher levels of depersonalization and personal accomplishment, whereas women exhibited higher levels of perceived stress and conscientiousness. Regarding nutritional habits, results were more positive for women, and men exhibited healthier functional habits by engaging in more weekly sports. Regarding oral health habits, women had better oral hygiene practices, brushing their teeth more frequently. However, women showed a higher tendency to smoke than their male counterparts. We conclude that there are notable gender differences that can provide insights for developing strategies to enhance the overall well-being of teachers.

## 1. Introduction

Stress is a response primarily regulated by the autonomic nervous system and the hypothalamic–pituitary–adrenal axis [1]. This response evolves to adapt to the demands imposed by an individual’s stimulating and ever-changing environment. The goal of this complex reaction is to maintain organic homeostasis through cardiovascular, metabolic, and immunological processes [2]. However, continuous exposure to a stressor leads to alterations in the autonomic nervous system, resulting in the onset of psychopathologies such as depression and anxiety [3,4,5]. Particularly in the workplace, and more specifically in the educational context, there is regular exposure to stressors. A study by Biron et al. found that 40% of teachers reported experiencing severe psychological distress, double the percentage in the general population (20%) [6]. This ongoing exposure and the consequences of unmanaged stress [7] can lead to burnout syndrome, a common psychopathology among this group [8], characterized by three dimensions: emotional exhaustion, depersonalization, and reduced personal accomplishment [9]. Therefore, it is possible to evaluate the emergence of this syndrome and the different personality traits that may predispose individuals to it through various questionnaires [10]. In academic settings, teachers often feel emotionally drained, exhibiting emotional and behavioral symptoms that can lead to disorders like depression due to personal, professional, and emotional demands [11,12,13], indicating these symptoms are linked to the inability to control work-related stress [7]. Burnout syndrome arises when there is a mismatch between job demands and uncontrolled stress, affecting performance in the short and long term. This can lead to the development of physical and mental illnesses, associated with about 50% of medical leaves [13,14,15,16] and a high rate of workplace absenteeism [13]. For common mental illnesses, a few days off may be granted, but according to 2018 statistics, they are the leading cause of work disability in Central Europe, thus posing a significant socioeconomic challenge in several European nations [17]. Consequently, due to the severe impact of work-related psychopathologies, various authors propose psychological and behavioral tools for effective stress management [18,19,20]. 

There is a multivariate component to enhancing individuals’ ability to modulate their stress response. Engaging in physical activity is identified as one of the most significant factors, as regular practice is associated with significantly lower levels of perceived stress [21,22]. This effect is due to the increase in neurotransmitters such as serotonin, dopamine, and endorphins during and after exercise, which are responsible for modulating the autonomic nervous system [23,24,25,26]. Additionally, mind–body activities like yoga are recommended for improved stress control [23]. Such modulations have been studied in other population groups, like the military, where a systematic review concluded that chronic autonomic and cardiovascular stress in soldiers appears to be managed through experience and prior training. However, the extent of this management may depend on the activity performed [27], thus explaining how controlled exercise modulates autonomic stress. Another important factor influencing the modulation of the stress response is nutrition; it is crucial in some studies since the consumption of carbohydrates, saturated fats, and refined sugars is linked with the onset of depressive states and stress [28,29,30,31]. This situation is exacerbated by a low intake of vegetables, dairy, and fruits [32,33]. Furthermore, studies demonstrate that nutrition is bidirectional, as increased calorie consumption is observed in individuals under stress [34,35,36]. However, recent research suggests that healthy eating is not linked with immediate stress modulation but may affect long-term well-being if there are physical consequences impacting the individual [37]. Finally, the oral health status of individuals is also associated with stress levels. It is observed that patients with high levels of perceived stress show alterations in their oral habits, leading to poorer dental hygiene and thus affecting periodontal tissues. Therefore, various studies indicate that the onset of periodontal diseases may be a consequence of patients experiencing conditions like stress [38,39]. On the other hand, dental problems are also linked to patients prone to mental illnesses, as studies have shown that people with functional oral or phonetic problems affect interpersonal relationships, leading to limited social activities and thus increasing feelings of isolation, loneliness, stress, and depression [40].

Due to the complex nature of psychopathologies manifested in the workplace, it is necessary to study the interactions between biological factors and social determinants, including gender stereotypes, as differences are expected in the establishment of socially described roles [41]. Meta-analysis studies have observed gender differences in the perception and communication of stress. These studies aim to resolve the role conflict assumed by each gender in their professional establishment, providing data where women score higher in emotional exhaustion and men show higher levels of depersonalization [42,43,44]. The above findings confirm theoretical claims that employees in gender-atypical occupations experience adverse health-related effects. Importantly, however, women employed in male-typed occupations experience negative outcomes for very different reasons than men employed in female-typed occupations. Women in male-typed occupations feel that they may be criticized for playing too feminine or not too feminine. Along with feelings of inferiority, the perceived and actual stressors associated with being a minority can create stress on women’s roles in male-type roles [45,46]. Though popular belief holds that women experience and are impacted by work–family conflict to a greater extent than men [47], the issue is more nuanced in reality: Byron’s meta-analysis demonstrated that women are more likely to experience family-to-work conflict, whereas men are more likely to experience work-to-family conflict [48]. Specifically, because women are still responsible for well over 50% of household chores and because they are still the primary child and elderly caregivers, they experience more family-related demands, such as role juggling and role conflict. Further, because men tend to be employed in white-collar jobs, they experience more job-related demands, such as time pressure and high expectations [49]. For this reason, we observe gender differences in stress management across various professions. In the hospital context, there is a significant increase in individuals with high levels of stress, burnout syndrome, and depression [50,51] due to the high psychosocial workload these individuals face [52]. We also found data in other research that female accountants scored highest on an anxiety measure and reported more work-related hassles compared to male accountants and male and female nurses. In contrast, male nurses had the highest rate of sickness absence compared to female nurses and male and female accountants and also perceived more work-related hassles than female nurses (vans and Steptoe, 2002). Furthermore, meta-analysis studies have found that women have a significantly higher risk of suicide compared to their male counterparts, with both genders exhibiting higher rates than those in the general population [50]. This gender difference can be explained by the additional tension imposed by social roles [53]. An additional aspect that deserves consideration is the complex and multifaceted relationship between creativity and personality. Research indicates the presence of certain personality traits in subjects with high creativity, and a more detailed understanding of these traits could be fundamental to understanding the differences in teaching behavior between genders within educational contexts in order to adequately address the needs of students. According to Plomin (2003), these characteristics include qualities such as a marked capacity for cooperation, remarkable persistence, and pronounced self-direction, which are representative of the teaching profile [54]. Effective management of these behaviors is essential for personality development and provides valuable insights into the various strategies for coping with situations in which personality plays a critical role in career success [55,56]. Gender influences the self-assessment of creativity, according to various studies, and the comparison between the two genders is vital to understanding the differences in creative thinking capacity. Kaufman found that men considered themselves more creative in scientific-analytical and athletic aspects, while women did so in aspects of social and visual–artistic communication [57]. Other studies showed significantly higher data for female college students on the originality subscales [58]. However, Henderson (2003) found no significant differences between the two genders in terms of creativity, suggesting that the relationship between creativity and personality is complex [59]. These discrepancies could be because self-assessments reflect internalized gender stereotypes rather than actual differences. Abra and Valentine-French (1991) propose that gender differences in creativity are explained by biological and environmental factors, such as specific cognitive abilities and educational opportunities [60]. Thus, creative achievement depends on both biological and environmental factors. So, we would argue that assuming any gender differences in creativity are most likely the product of differing environments would represent the best overall synthesis of what we currently know about gender differences in creativity [57]. Other gender differences were observed during the COVID-19 pandemic, which affected men and women differently. The authors have suggested that there is a gender difference in the psychological experience, somatization, and impact of the COVID-19 pandemic and the emotions it provokes, suggesting that women are more emotionally vulnerable to the effects of the COVID-19 context than men [61]. While men had higher fatality rates and a greater risk of mortality [62], women showed a higher prevalence and severity of symptoms of anxiety, depression, and acute stress [63]. This may be related to the greater levels of state-trait anxiety reached in this study, where females presented higher levels than males in line with previous literature [64]. Additionally, during the pandemic, significant psychological effects were observed in students and teachers due to academic and job uncertainties, leading to depressive symptoms affecting these groups [65]. In these studies, female students exhibited a higher prevalence of depression than their male counterparts [66]. In addition, psychometric analysis reveals that variations in personality traits between genders could be responsible for the influence of perceived risk and anxiety in the female gender. According to the data obtained, it is observed that male students exhibit higher levels of extraversion compared to their female counterparts. On the other hand, female students show higher rates of conscientiousness and neuroticism [67]. These findings are consistent with existing literature indicating greater openness to new experiences in women, which contrasts with the results obtained by Castañeiras et al. (2006), who found greater openness in men [68]. However, these discrepancies could be explained by differences in sociocultural contexts (Latin America–Europe) and the particularities of the samples studied.

Other Possible sex differences in work-related stress are due in part to the different types of work carried out by men and women. In the UK, women are concentrated in clerical, service, and sales work and in jobs such as teaching and nursing (Office for National Statistics, 1998) [69]. Women earn less than men and occupy less prestigious positions at work [70]. In 1993, men were twice as likely as women to be managers and administrators. Women experienced lower control and higher job strain than men [71] and had fewer learning opportunities at work [72]. Emslie et al. (1999) have argued that when men and women in the same occupation are compared, sex accounts for little of the variance in psychological distress or minor morbidity in comparison with working conditions. However, this pattern may depend on the culture of the workplace [70].

Regarding teachers, previous studies have noted gender differences in stress response, with female teachers exhibiting higher levels of emotional exhaustion [73] and perceived stress [74] and a greater prevalence of burnout syndrome compared to men [73,75]. However, other authors have found no statistically significant gender differences in perceived stress [75]. Given the multifactorial basis and the controversy found in the scientific literature, it is interesting to investigate consistent findings that clearly conclude the influence of gender on the onset of stress and exhaustion in teachers. Therefore, we present an in-depth analysis of behavioral differences in psychological, nutritional, physical activity, and oral health factors related to stress. Hence, we propose the following study with the objective of analyzing gender differences in psychological, nutritional, physical activity, and oral health factors that are associated with stress levels in teachers with the aim of developing specific and effective strategies to promote their overall well-being. The initial hypothesis was that there would be gender differences in both the stress-related factors experienced by teachers and the psychological, nutritional, physical activity, and oral health components.

## 2. Materials and Methods

### 2.1. Participants

A study was conducted using an online sample during the academic year 2020–2021, consisting of 1031 active teachers from Spain (89.9%), Colombia (7.4%), and Chile (2.6%). This sample comprised 413 men and 618 women, aged between 20 and 70 years, with an average age of 41 years for men (41.5 ± 9.5 years) and 41 years for women (41.3 ± 8.9 years). Regarding their teaching level, 19.7% worked in primary education, 13.6% in secondary education, 56.8% in higher education institutions, and 6.3% in vocational/professional training. Concerning teaching experience, an average of 11 years (11.8 ± 9.2 years) was observed in men and 12 years (12.2 ± 8.7 years) in women.

The selected subjects voluntarily participated in the study by completing an online survey. The respondents were previously informed about the study and gave their informed consent, ensuring confidentiality and anonymity in accordance with the Organic Law on Data Protection (BOE-A-2018-166673). Prior to taking the survey, all participants completed an informed consent form, a procedure authorized by the university’s ethics committee, assigning it an internal code of CIPI/22.178. All procedures were carried out in accordance with the Helsinki Declaration (as amended in Brazil in 2013).

### 2.2. Design and Procedure

To achieve the results of this research, an analytical, observational, cross-sectional, and retrospective study was conducted. The study sample was obtained through a survey conducted on the selected population using an online platform via Google Docs, with the variables shown below. The survey offered binary and multiple-choice responses about the respondents’ self-perceived health. The studied variables listed below were examined based on previous research.

#### 2.2.1. Anthropometric Variables

Participants measured their height and weight and entered the results in the questionnaire. These variables were considered for the analysis of the Body Mass Index (BMI). 

#### 2.2.2. Variables Related to Teaching Activity and Psychological Profile

The following variables were analyzed to test variables related to teaching activity.

- Teaching Experience: An objective scale representing the number of years of professional experience in teaching.
- Teaching Satisfaction: Measured on a self-perception scale regarding how satisfied the teacher feels with their current teaching role. This item is a Likert-type scale or rating scale from 0 to 10, where 0 indicates not satisfied at all and 10 indicates completely satisfied.
- Teacher Stress: A self-perception of current stress using a Likert-type or rating scale from 0 to 10, where 0 indicates no stress and 10 indicates severe stress.

#### 2.2.3. Descriptive Variables Related to the Psychological Profile Using Validated Questionnaires in Spanish

- Emotional Exhaustion: The Spanish version of the Maslach Burnout Inventory Test (MBI) [10,76] was used. This test evaluates depersonalization, emotional exhaustion, and personal accomplishment as the three emotional components to assess the existence of burnout syndrome in the workplace. The test consists of 22 items, each rated on a Likert scale from 0 to 6. The Cronbach’s alpha coefficient for internal consistency reliability was 0.76 for depersonalization, 0.76 for exhaustion, and 0.90 for personal accomplishment. Examples of these items include: “Due to my job, I feel emotionally exhausted”; “I have an insensitive treatment with my students”; and “At work, I feel that I am at the limit of my possibilities”.
- Perceived Stress (PSS) [77,78]: This test involves a self-assessment of current perceived stress using 14 elements. The test consists of 10 items with response possibilities from 0 to 4 on a Likert scale, where 0 means “never” and 4 means “very often”. Higher scores indicate that the person is experiencing a high level of perceived stress along with a loss of control. Examples of these items include: “I have lost control of important things in my life”; “In the last month, I have frequently been upset by something unexpected”.
- Psychological Inflexibility: The Spanish version of the Acceptance and Action Questionnaire (AAQ-II) is the most commonly used measure of psychological inflexibility [79]. It is a test that measures the capacity to handle unpleasant internal emotions or situations through seven items on a 7-point Likert scale, where 1 is “never” and 7 is “always”. Scores range from 7 to 49. Higher scores indicate that the individual has the capacity to control aversive thoughts or feelings. The Cronbach’s alpha coefficient for internal consistency reliability was 0.93 for men and 0.95 for women. Examples of these items include: “I worry about not being able to control my worries and feelings”; “Worries get in the way of my success”.
- Loneliness: Spanish version of the UCLA Loneliness Scale [80]. It assesses the self-concept of loneliness as well as the feeling of being excluded and/or receiving less social support than desired. It is rated on a 3-item scale with responses on a scale of 1 to 3, where 1 is “almost never,” 2 is “sometimes,” and 3 is “often”. Scores range from 0 to 9. Lower scores indicate a greater feeling of loneliness and/or no social support. The Cronbach’s alpha coefficient for internal consistency reliability was 0.76 for men and 0.84 for women. Examples of these items include “I feel excluded” and “I feel isolated from others”.
- Big Five Personality Test: Abridged Spanish Version of the Big Five-Factor Inventory [81]. This assessment evaluates five personality dimensions: neuroticism, extraversion, agreeableness, conscientiousness, and openness to experience, using 10 items from the original 44. Each dimension is rated on a Likert scale ranging from 1 (“strongly disagree”) to 5 (“strongly agree”). For internal consistency reliability, the Cronbach’s alpha coefficient was 0.75. Sample items include: “I often find faults in others,” “I am reserved,” “I consider myself sociable,” “I see myself as persevering,” and “I think of myself as highly imaginative”.

#### 2.2.4. Descriptive Variables Related to Nutritional Habits 

Previously utilized questionnaires [82,83,84] were adapted for this study. Two sets of queries were employed:

- First set: Focusing on individual behaviors and perceptions regarding nutrition, e.g., “Do you believe you have a healthy diet?”; “Do you eat between meals?”; “Do you follow a diet?”; “Do you read nutritional information when shopping for food?”; “Do you eat slowly and while seated?”. These five items had dichotomous responses, with 0 representing ‘no’ and 1 ‘yes’.
- Second set: Concerning the weekly frequency of consuming various food groups (ranging from 1 to “more than 5 times per week”), such as fruits/vegetables, meat, fish, legumes, fast food, snacks, fried foods, sodas, and distilled beverages. This section also included daily consumption frequency questions about dairy products, water, and meals, as well as inquiries regarding daily sleep hours.

#### 2.2.5. Variables Related to Physical Activity 

These variables were analyzed using questionnaires previously employed by other researchers [28,82]. Participants reported their physical activity behaviors and, if applicable, the amount of time dedicated daily and weekly to these activities (ranging from 1.5 h to “more than 12 h per week”). This included time spent on non-organized sports, endurance sports, team sports, and weekly training sessions. Additionally, questions about the number of sick days per year were included.

#### 2.2.6. Variables Related to Oral Health 

These variables were assessed using a previously established questionnaire [82,83]. The questionnaire consists of 6 items pertaining to oral health habits and the individual’s perception of certain symptoms.

- First set: Focused on individual perceptions of specific oral health symptoms. These four items had responses ranging from 0 (‘no’) and 1 (‘sometimes’) to 2 (‘yes’), including queries such as “Do you believe you have bad breath?”; “Do your gums bleed?”; “Do you frequently experience dry mouth/lack of saliva?”; and “Do you suffer from gastritis or heartburn?”.
- Second set: Concerned individual habits related to oral health, such as “Do you smoke?” and “How many times do you brush your teeth per day?”. These items assessed the frequency of these habits, with responses varying from 0 to “more than 4” for daily tooth brushing and from 0 to “more than 5 cigarettes per day” for smoking habits.

### 2.3. Statistical Analysis

Statistical analyses were conducted using the Statistical Package for the Social Sciences (SPSS) version 24.0 (SPSS Inc., Chicago, IL, USA). Descriptive statistics (mean and standard deviation) were calculated for each variable. The Kolmogorov–Smirnov tests were applied to assess the normality and homogeneity of the variables. Independent *t*-tests were employed to examine gender differences. A bivariate correlational analysis was conducted using Pearson’s correlation test. The significance level for all analyses was set at *p* ≤ 0.05.

## 3. Results

This study analyzed data from 1031 active teachers, comprising 40.1% males and 59.9% females, with an average age of 41 years (41.5 ± 9.5 years for males and 41.3 ± 8.9 years for females) and an average teaching experience of 11 years (11.8 ± 9.2 years for males and 12.2 ± 8.7 years for females).

Regarding psychological profile data, male teachers exhibited significantly higher levels of depersonalization (*p* = 0.000) and personal accomplishment (*p* = 0.043) compared to their female counterparts. Conversely, female teachers demonstrated significantly higher levels of perceived stress (*p* = 0.001) and conscientiousness (*p* = 0.014) than male teachers. No statistically significant gender differences were found in teacher satisfaction, teaching stress, emotional exhaustion, psychological inflexibility, loneliness, extraversion, agreeableness, neuroticism, and openness to experience (Table 1).

In terms of nutritional habits, male teachers exhibited significantly higher figures in the number of glasses of water consumed daily (*p* = 0.000), the tendency to snack between meals (*p* = 0.025), adherence to diets (*p* = 0.000), and weekly intake of legumes (*p* = 0.022), fried foods (*p* = 0.000), and distilled beverages (*p* = 0.000). On the other hand, female teachers showed significantly higher levels in the number of meals per day (*p* = 0.027), the practice of eating slowly (*p* = 0.000), daily dairy consumption (*p* = 0.032), and weekly fish consumption (*p* = 0.000). No statistically significant gender differences were observed in other nutritional values (Table 2).

According to the physical activity data, significantly higher values in unstructured sports participation/minutes of daily movement were found among female teachers (*p* = 0.009). However, the figures for participation in endurance sports (*p* = 0.000), team sports (*p* = 0.000), and resistance training (*p* = 0.000) were significantly higher in males. No gender differences were observed in the incidence of illness per year (Table 3).

Regarding oral health habits, female teachers showed significantly higher incidences of dry mouth (*p* = 0.000), gastritis (*p* = 0.000), smoking habits (*p* = 0.000), and frequency of daily tooth brushing (*p* = 0.011). In contrast, no significant data were observed for the male gender. No statistically significant differences were found in issues related to gum bleeding and halitosis (Table 3).

## 4. Discussion

The objective of this study was to analyze gender differences in psychological, nutritional, physical activity, and oral health factors that are associated with stress levels in teachers in order to develop specific and effective strategies for promoting their overall well-being. The initial hypothesis was partially confirmed as statistically significant differences between genders were observed. In the male gender, significantly higher data were noted in depersonalization, personal achievement, diet adherence, the habit of eating between meals, daily water intake, consumption of legumes, fried foods, and distilled beverages per week. Additionally, higher values were found in engaging in endurance sports, team sports, and overload training per week. On the other hand, significantly higher values were observed in the female gender in perceived stress, conscientiousness, the habit of eating slowly, the number of meals per day, the amount of fish consumed weekly, and the number of dairy products consumed daily. High values were also found in smoking habits, number of teeth brushings per day, gastritis, dry mouth, and daily movement with non-regulated sports. No statistically significant differences were observed in the rest of the parameters studied.

In the three dimensions that make up the Maslach Burnout Inventory, in our research, we found significant gender differences in depersonalization and self-fulfillment. The results indicate that males obtained higher levels of depersonalization, an alteration of the consciousness of the self, in such a way that the depersonalized individual feels himself as strange and distant, a mere observer of his mental processes and his bodily sphere [85]. These results are supported by other research, which also shows higher levels in males than in females [86,87]. These findings are in line with previous findings that reported an increase in these aspects among men [88]. Improving the quality of life for both male and female workers is imperative, as safe working conditions contribute greatly to overall well-being [89]. Burnout syndrome, characterized by professional exhaustion, depersonalization, and low personal accomplishment, is prevalent among professions involving intense human interaction, such as teaching [90]. While some studies [91,92,93] found higher depersonalization scores among female teachers compared to male teachers, others indicated higher levels of depersonalization and personal fulfillment among male teachers. This highlights the need for more research to investigate these contradictory gender differences in teacher burnout. In this research, countries from South America and the European Union (EU) were selected due to the similarity in social policies, particularly after the consolidation of the EU. A comparative analysis of employment policies reveals a divergence between the United States and the nations examined in this study, indicating that the former maintains a more conservative approach to labor legislation, while the EU and the mentioned South American countries tend towards a more socially progressive stance. Previous research has corroborated these results in relation to depersonalization, showing that gender disparity in this phenomenon is more pronounced in the U.S. compared to more progressive labor policies. Specifically, it is likely that males in the U.S. report experiences of depersonalization compared to the average woman in the same country. In contrast, in contexts where labor policies are more advanced—in EU member states and certain South American countries—gender differences in terms of depersonalization are smaller [94]. Even more relevant, these findings offer initial support to the hypothesis proposed by various entities and governmental organizations, which assert that labor policies with a socially responsible approach are essential for the well-being of workers, as suggested by the International Labour Office in its 2008 report [95]. Additionally, our study observed significantly higher levels of perceived stress among females. Teaching, a profession prone to high stress levels, often exceeds that of other high-risk professions [96], and this is consistent with previous studies showing higher stress levels in female teachers [97,98,99]. The dual burden of work and home responsibilities for women could be a contributing factor, leading to heightened stress levels compared to men, who generally face fewer domestic demands [100]. The interaction between work and family life is significantly correlated with occupational stress [101].

Looking at other collectives than teachers, we find similar results, as male workers in the health sector or in the banking sector also showed higher levels than females [102,103,104]. Regarding self-fulfillment, we found that females present lower levels than males. In line with this, a recent study shows comparable results among university professors [67]. Likewise, in the present study, females presented higher levels of perceived stress. Previous authors also found higher levels of perceived stress values in female than male Spanish college professors [105]. Not only in the Spanish population but also in Portuguese and American populations, as the previous bibliography points out [42,67,106]. Regarding personality traits that may influence perceived stress, in our study, we found higher levels of conscientiousness in females than in males and no significant differences in extroversion, agreeableness, neuroticism, or openness to experience. Reviewing the previous literature and analyzing other university groups, such as students, we found results where females also presented these levels [107].

Our study revealed a notable aspect: although women reported higher levels of perceived stress compared to men, this increased stress did not translate into differences in teaching stress or teaching satisfaction. This finding suggests that, despite facing greater general stress, female educators maintain a level of resilience or coping mechanisms that enable them to manage teaching-related stress effectively and derive satisfaction from their roles similar to those of their male counterparts. It raises intriguing questions about the underlying factors contributing to this resilience and satisfaction in teaching, despite higher overall stress levels in women. Further investigation into these dynamics could provide valuable insights for developing targeted support and interventions in educational settings.

As for nutritional habits, males in our study showed a higher adherence to diets. Prior research indicated a higher prevalence of overweight among Spanish adult males (43.9%) [108], an issue of growing concern in public health globally [109]. Higher consumption of fried foods was also noted among male teachers. A recent study found that high consumption of fried foods adversely affects anxiety and depression due to altered lipid metabolism and neuroinflammation [110]. Furthermore, our study observed a higher water intake among male teachers, aligning with the European Food Safety Authority (EFSA) guidelines suggesting greater hydration and nutritional needs in men compared to women [111]. Higher levels of distilled beverage consumption were also observed among male teachers. Limited research exists on teachers’ consumption habits and their consequences, as harmful alcohol use in educational settings is typically associated with students [112]. Alcohol consumption has been reported as a coping mechanism for personal problems and crises [113], indicating the need for future research to include teachers in health promotion and preventive campaigns in educational settings related to alcohol consumption. In contrast, female teachers showed a tendency to eat more slowly, a practice scientifically proven to reduce food and energy intake [114] and potentially protect against overeating and conditions like overweight or obesity [115]. Additionally, female teachers reported having more meals per day. Stress activates the hypothalamic–pituitary–adrenal axis, increasing glucose demand for bodily homeostasis and thus elevating appetite and food intake [116]. Numerous studies have examined the relationship between stress and eating behavior, leading to the concept of ‘emotional eating,’ which refers to the use of food as a dysfunctional coping mechanism for negative emotions [117,118].

Regarding physical activity, male teachers demonstrated higher levels of physical activity than their female counterparts. Higher physical activity levels are associated with better control in stressful situations [119]. In Spain, gender differences persist in sports participation, with higher rates among men (63.1% annually) compared to women (51.8%) and a growing preference for individual sports [120]. Male teachers engaged more in resistance training, directly influencing health, hence its inclusion in health improvement and quality of life programs [121]. Female teachers participated more in unregulated sports activities, highlighting the importance of promoting women’s sports for healthy lifestyle habits [122]. Individuals with sedentary lifestyles tend to smoke more, aligning with our findings, where less physically active women were more likely to smoke [123]. In terms of oral health habits, female teachers exhibited significantly higher levels of dry mouth, gastritis, and daily tooth brushing. Generally, being female is associated with better oral health habits [124]. Higher levels of dry mouth in female teachers, related to stress, anxiety, and smoking, were consistent with our study’s findings [125]. Higher instances of gastritis in female teachers align with other studies finding elevated gastritis levels in academic teachers regardless of gender [126].

## 5. Conclusions

In conclusion, this study successfully achieved its objective of examining gender differences in psychological, nutritional, physical activity, and oral health factors associated with stress levels in educators. The research revealed significant gender disparities, with higher levels of depersonalization and personal fulfillment observed in males and greater perceived stress in females. Additionally, notable differences in nutritional habits and physical activities were identified between genders. These findings provide essential insights for developing targeted strategies to enhance the overall well-being of educators, highlighting the importance of considering gender-specific needs in stress management and wellness initiatives.

## 6. Practical Applications

The findings of our study have several practical applications, particularly in educational and occupational health settings. Firstly, they underscore the importance of implementing targeted stress management and wellness programs in schools tailored to address the specific needs of male and female educators. Secondly, this study highlights the need for comprehensive health promotion strategies in the workplace that include nutritional guidance and physical activity encouragement, addressing gender-specific health behaviors and preferences. Lastly, our research suggests the value of including mental health and resilience training as part of professional development for educators, considering the different stressors and coping mechanisms observed between genders. These applications aim to enhance the overall well-being and job satisfaction of teachers, contributing to a healthier and more productive educational environment.

## 7. Limitations of the Study and Future Lines of Research

One of the limitations of our study is the specific demographic and geographic makeup of our sample, which may affect the generalizability of our findings. Future research should aim to include a more diverse participant pool from various regions and backgrounds to enhance the applicability of the results. Additionally, the cross-sectional nature of this study limits our ability to draw causal inferences. Longitudinal studies would be beneficial to understand the dynamics of stress and wellness over time. Moreover, incorporating the use of heart rate variability (HRV) as an objective measure, in line with other studies in the educational field [11,127,128,129], would provide valuable insights into the physiological aspects of stress among teachers. This approach aligns with recent trends in stress research that emphasize the importance of physiological markers. Finally, our research primarily relied on self-reported measures, which may be subject to bias. Future studies could incorporate more objective data collection methods, including physiological stress markers like HRV, to provide a more comprehensive understanding of the phenomena under investigation.

## Figures and Tables

**Table 1:** Gender differences in psychological profiles.

Variable	Male	Female	t	Sig
Teaching satisfaction	8.4 ± 1.3	8.2 ± 1.3	0.158	0.691
Teacher stress	5.8 ± 2.3	6.9 ± 2.3	1.377	0.241
MBI. Emotional exhaustion	16.8 ± 9.7	20.1 ± 10.0	1.669	0.197
MBI. Depersonalization	4.7 ± 4.4	3.6 ± 3.7	14.634	0.000
MBI. Personal accomplishment	37.9 ± 6.2	36.7 ± 5.7	4.106	0.043
PSS. Stress perceived	19.9 ± 4.0	22.0 ± 4.5	10.696	0.001
AAQII. Psychological inflexibility	15.4 ± 8.5	16.2 ± 8.6	0.619	0.432
UCLA. Loneliness scale	3.8 ± 1.4	4.0 ± 1.5	1.377	0.241
NEO FFI. Extraversion	4.9 ± 1.8	4.7 ± 1.8	0.015	0.903
NEO FFI. Agreeableness	7.3 ± 1.6	7.2 ± 1.5	0.454	0.501
NEO FFI. Conscientiousness	8.0 ± 1.7	8.3 ± 1.5	6.138	0.014
NEO FFI. Neuroticism	4.7 ± 2.0	5.6 ± 2.0	1.002	0.317
NEO FFI. Open to experience	7.9 ± 1.8	7.9 ± 1.7	0.732	0.393

**Table 2:** Analyzed nutritional variables.

Variables	Male	Female	t	Sig
Hours of sleep per day	6.7 ± 0.9	6.8 ± 0.9	1.022	0.312
Healthy food	1.2 ± 0.4	1.2 ± 0.4	0.001	0.971
Snack between meals	1.547 ± 0.498	1.508 ± 0.500	5.060	0.025
Adherence to diets	1.9 ± 0.3	1.8 ± 0.4	97.224	0.000
Eating slowly	1.3 ± 0.4	1.3 ± 0.5	22.180	0.000
Number of meals per day	3.8 ± 0.9	4.0 ± 0.9	4.919	0.027
Number of glasses of water daily	3.1 ± 2.7	2.5 ± 1.9	12.791	0.000
Daily dairy	2.0 ± 1.2	2.3 ± 1.3	4.607	0.032
Weekly sweet consumption	1.2 ± 0.9	1.1 ± 1.0	0.007	0.932
Weekly meal consumption	3.2 ± 1.4	3.1 ± 1.4	2.182	0.140
Weekly fish consumption	2.1 ± 1.1	2.4 ± 1.3	26.620	0.000
Weekly legumes consumption	2.4 ± 1.2	2.0 ± 1.2	5.299	0.022
Fast food weekly	1.0 ± 1.0	0.9 ± 0.9	0.824	0.364
Fizzy drinks weekly	1.3 ± 1.7	1.3 ± 1.8	0.016	0.898
Fried foods weekly	1.5 ± 1.2	1.1 ± 1.1	18.645	0.000
Weekly fruits consumption	2.5 ± 1.6	3.0 ± 1.8	0.846	0.358
Distilled beverages weekly	1.3 ± 1.6	1.0 ± 0.2	37.851	0.000

**Table 3:** Variables in oral health habits and physical activity.

Variables	Male	Female	t	Sig
Daily Movement Time	1.7 ± 1.3	1.7 ± 1.4	6.798	0.009
Weekly Endurance Sport	1.4 ± 1.6	1.0 ± 1.3	25.529	0.000
Team Sport per Week	0.4 ± 1.0	0.1 ± 0.5	148.969	0.000
Overload Training per Week	1.0 ± 1.3	0.5 ± 1.0	49.881	0.000
Sick Days per Year	5.7 ± 18.4	7.1 ± 18.4	1.602	0.206
Times of Brushing Teeth per Day	2.6 ± 0.9	2.7 ± 0.8	6.518	0.011
Bleeding Gums	0.4 ± 0.6	0.4 ± 0.6	0.429	0.513
Bad Breath	0.3 ± 0.5	0.3 ± 0.5	0.308	0.579
Smokes	0.2 ± 0.7	0.3 ± 0.8	25.622	0.000
Dry Mouth, Lack of Saliva	0.4 ± 0.6	0.6 ± 0.7	36.747	0.000
Gastritis, Heartburn	0.3 ± 0.5	0.4 ± 0.6	44.172	0.000

## Data Availability

All data are presented in the manuscript.
